# A Study of Oddity in a Russian Clinical Sample

**DOI:** 10.11621/pir.2021.0102

**Published:** 2021-03-20

**Authors:** Julia A. Atadzhykova, Sergey S. Enikolopov

**Affiliations:** a Mental Health Research Center, Moscow, Russia

**Keywords:** oddity, schizotypy, schizophrenia-spectrum disorders, peculiar speech, *Verschroben*, psychological assessment

## Abstract

**Background:**

At the beginning of 20^th^ century, the phenomenon of oddity began to be studied. It was defined as a set of characteristics responsible for an individual giving the impression of being unusual, odd, and peculiar. Later, psychiatrists integrated oddity into the concept of schizotypy. Yet, while considered a part of the schizotypy construct, oddity has remained singular and maintained its status as an independent dimension.

**Objective:**

The present article discusses oddity as a set of particular clinical traits that can be evaluated both by self-report measures and clinical assessment. We set out to investigate the oddity phenomena as manifested in a clinical sample, in order to delineate key features that constitute this concept.

**Design:**

Seventy-one patients were selected according to a specific set of criteria and subjected to a set of self-report measures (the Schizotypal Personality Questionnaire and the Adult Personality Traits Questionnaire), a clinical interview, and a pathopsychological experiment. A number of cognitive, behavioral, and emotional characteristics were analyzed. An intra-group comparison was carried out in order to clarify the potential differences between the self-reported and clinically assessed phenomenon of oddity.

**Results:**

The study’s first finding was that the SPQ-74 does not identify odd personalities in the general population, as reflected in the fact that the sample’s average scores proved to be low. Secondly, restricted emotionality and a deficit in social interactions proved to be the prevalent characteristics of the sample of “odd” individuals. Furthermore, a set of certain speech peculiarities (word coinage, bizarrerie, etc.) and thinking impairments of various types (distortion of abstraction level and motivational deficit) emerged as prominent characteristics in the majority of subjects. Finally, it was determined that clinical assessment allows for a more comprehensive evaluation of the psychology of odd personalities than self-report measures, due to a number of the personality, temperamental, and cognitive characteristics that the latter tend to exhibit.

**Conclusions:**

“Odd” individuals can be characterized by a number of cognitive, emotional, and behavioral features independent of social perception and relevant to clinical practice; they can be captured more successfully by the application of qualitative methods. Further research is needed to elaborate this set of traits and test this hypothesis on new samples.

## Introduction

The awareness that a specific group of individuals can be distinguished by a set of peculiarities that manifest through a variety of behavioral patterns became the focus of attention many years ago, back at the beginning of the 20^th^ century. Clinicians of that period noted that a number of individuals on the schizoid spectrum demonstrated various cognitive, emotional, and behavioral qualities that often appeared strange, odd, and unusual to the public eye. Kraepelin (1915) described so-called “eccentric personalities” based on his observations that the relatives of schizophrenic patients showed attenuated signs or mild forms of impairments characteristic of schizophrenia itself. These individuals’ peculiarities involved ambivalence and incongruence of emotionality, absurd ideas (*e.g.*, ludicrous dieting), disintegration of thought, vague speech, etc.

In addition, he separated out a group of “odd personalities”, whom he put in the category of psychopaths, potentially qualifying this phenomenon as distinct from endogenous disorders.

Birnbaum (1920) identified opposition to society as a common feature of oddity, whether it expressed itself outwards or was projected inwards, and highlighted cognitive characteristics such as rigidness, and non-continuous, or autistic, thinking. Binswanger (1954) was the first author to use the German word “odd,” and differentiated the two clinical contexts where it had been exploited: 1)*verschrobenen Psychopathen*(ger., “odd psychopaths”), which indicated a group of individuals who supposedly shared a personality disorder; and 2)*schizophrenen Verschrobeneheit*(ger., “schizophrenic oddity”), a strangeness that resulted from an endogenous disorder. Importantly, Binswanger pointed out that oddity represented an aspect of personality rather than an isolated symptom.

Later, when Rado (1953) proposed the term “schizotypy,” oddity became an inherent part of the formulation of the construct. Consequently, the research continued within the framework of schizotypy studies. Two chief research directions were delineated: familial and clinical. The former line of studies focused on the observations and investigation of the relatives of schizophrenic patients, among whom various anomalies of character are often present. Analysis of the descriptive works in this line of research (Eysenck & Barrett, 1993; Kendler, 1985) identifies five characteristics that were often prominent in this group of individuals: 1) eccentricity; 2) irritability; 3) social isolation; 4) aloofness; and 5) suspiciousness. These characteristics can be considered the definition of the concept of schizotypy.

Within clinical studies, oddity has been investigated in the context of mental illnesses such as schizophrenia. Rado (1953) described schizotypy as a “psychodynamic manifestation of schizophrenic genotype,” a notion that was later developed by Meehl (1962), who used the term “schizotaxia’ to refer to an integral neural deficit. According to Meehl, a particular genetic basis, which interacts with various environmental factors such as social learning, education, etc., creates a schizotypic personality organization. Although this personality predisposition is considered a risk factor for developing schizophrenia, Meehl notes that the “stress-diathesis” model allows different developmental pathways, thus highlighting the fact that not all schizotypic individuals will develop schizophrenia. Meehl proposed four core symptoms to constitute schizotypy: anhedonia; social aloofness; cognitive slippage; and ambivalence. Thus the concept of schizotypy is phenotypically similar to the schizotypal personality disorder as formalized in DSM-III, the chief distinction being the fundamental difference in their origins (APA, 1980; Bove, & Epifani, 2012).

Russian clinicians also followed the clinical tradition and conceptualized oddity phenomena in line with the traditional medical model (Smulevich, 2016; Snezhnevsky, 1983; Vorobyov, 1988). Relying on the works of Kraepelin (1915) and Bleuler (1993), Russian psychiatrists developed the notion of the*Verschroben*-type schizophrenic defect, which was categorized as psychopathy-like negative changes and manifested through odd, extravagant behavior, strange interests and hobbies, peculiarities of movement and speech, etc. (Snezhnevsky, 1983; Vorobyov, 1988). Then the research of oddity per se became more or less neglected, since strangeness was either considered a part of a more extensive construct of schizotypy or formulated within the context of endogenous disorders.

Soon after Meehl’s proposed schizotypy model, other authors deemed it reasonable to bring forward an alternative dimensional model, which broadened the clinical context of this construct (Claridge et al., 1996). In these works, schizotypy was conceptualized as representing both a predisposition to developing psychosis, and a source of individual differences in general population. The understanding of schizotypy as a dimension allowed it to be presented in a continuous form – as a multifactorial construct. The authors formulated four core dimensions that were seen as inherent to schizotypy; these were 1) unusual experience, 2) cognitive disorganization, 3) introversive anhedonia, and 4) impulsive non-conformity (Corcoran, Devan, Durrant, & Liddle, 2013).

The idea that oddity can be observed in a normal personality stimulated attempts to distinguish the oddity domain in the multifactorial personality model (Widiger, 2010; Verbeke & De Clercq, 2014). In that model, oddity is perceived as a core personality dysfunction specific to A-cluster personality types. Verbeke and colleagues found statistical and empirical grounds for the existence of this fifth factor in a sample of over 400 adolescents. The oddity factor featured hypersensitivity to feelings, excessive fantasizing, daydreaming, and unusual thoughts and behavior. The notion that A-cluster personalities, and schizotypal individuals in particular, are seen as strange and eccentric, provides evidence for the strong relevance of oddity features for schizotypy and schizoid-spectrum phenomena, whereas the continuous nature of schizotypy also allows for the occurrence of oddity features in normal personality (Ashton & Lee, 2012; Grant, 2015; Lenzenweger, 2015).

All in all, the research at the beginning of the 20th century provided descriptive studies of oddity, wherein particular traits or impairments characteristic of these individuals were defined. Later, this set of peculiarities was transformed into the notion of schizotypy, which merged oddity with other characteristics of schizophrenia-spectrum disorders. Conspicuously, schizotypal individuals have mostly been described as “strange” and “unusual,” so that these everyday epithets made their way into the official formulation of the schizotypal personality disorder (APA, 1980; 2013). Analysis of the body of work dedicated to the issue of oddity allows one to delineate a number of key features that may be definitive for oddity itself. They are: 1) the impairment of social functioning; 2) sensitivity; 3) eccentricity; and 4) peculiarities of cognitive processes. (Chemerinski, Triebwasser, Roussos, & Siever, 2013; Cohen & Lee, 2011; Fumero, Rodríguez, Roa, & Peñate, 2017; Verbeke, De Clerq, Van der Heijden, Hutsebaut, & van Aken, 2015).

In western clinical science, oddity has been operationalized as “perceived strangeness or eccentricity” (Ashton & Lee, 2012). In this frame of reference, the oddity phenomenon is mostly studied psychometrically, and its core notion is explained and/or defined with the help of a variety of common adjectives, such as “bizarre,” “peculiar,” “unusual,” and some others, including the negatives like “not average,” “not normal,” etc. This approach involves social perception as the chief factor that is supposed to clarity the content of these phenomena. However, it does not necessarily shed light on the problem of clinically assessing the oddity phenomenon. For example: If changing occupation had been considered unusual and often maladaptive in Russia a decade ago, it is more likely to be viewed as a flexible and healthy way of life in 2021. Such shifts in social perception of what is “normal” and acceptable behavior leave little opportunity for practicing clinicians to identify, and even less operationalize, the oddity phenomenon.

In modern Russian psychiatry, oddity is studied under the name of “*Verschroben*”-type negative changes in schizophrenia-spectrum disorders (Mukhorina, 2018; Smulevich, Romanov, Mukhorina, & Atadzhykova, 2017). This line of research is mainly grounded in studies of schizotypy as a categorical construct, and traditional Russian clinical studies of schizophrenia – namely, the*Verschroben*-type defect (Smulevich, 2016; Snezhnevsky, 1983). This medical model of the*Verschroben* syndrome considers endogenous factors as determinant of the development of oddity.

In our study, we have proposed a set of manifestations of this syndrome, which include: 1) distinctness of behavior that is not congruent with the social and cultural context of one’s life; 2) impairment of social functioning; 3) emotional deficit such as coldness and/or blunted affect; 4) occupational dysfunction; and 5) unusual perceptions of one’s body, distorted bodily image, and or/ psychosomatic disorders (Smulevich et al., 2017). As can be seen, this list includes cognitive, emotional, and behavioral patterns that move beyond the criterion of social perception, which allows more clarity and flexibility for qualitative study. In this modern conceptualization, the notion of*Verschroben* serves to absorb the data accumulated in different contexts of studying the oddity phenomenon, thus acquiring clinical value that is expected to prove useful both in theory and practice today.

The present article discusses the results of a psychological study of patients with diagnozed*Verschroben* syndrome. The investigation has been carried out in collaboration with the Russian psychiatrists and is theoretically based on a vast field of prior research (for more extensive theoretical reviews, see Atadzhykova & Enikolopov, 2016; Mukhorina, 2018).

## Methods

### Participants

The clinical sample included 71 patients (44 women and 27 men, average age = 40.2) who were selected and examined at the premises of the Department of Borderline Mental Pathology and Psychosomatic Disorders of the Mental Health Research Center (led by the academician of RAMS, A. Smulevich) and the Department of Psychotherapy of the Clinical Centre of Psychosomatic Medicine of Sechenov University in Moscow. Subjects were included in the clinical sample if they had previously received a F21 diagnosis (for more details, see Mukhorina, 2018). The sample was formed after the subjects were examined by a council of clinicians which agreed that they met the examination criteria, and had medical histories which included years-long records of unusual, bizarre, and/or peculiar behavior. Due to the absence of a control group, various sub-groups (*e.g.,* with highest/lowest SPQ scores) were selected from the clinical sample for further intra-group comparison.

### Procedure

The chief goal of this study was to analyze the cognitive and personality characteristics of patients who demonstrate the phenomenon of oddity. The study’s tasks included: 1) specification of the emotional and personality characteristics that may be relevant to the differentiation between self-report and clinically assessed oddity; 2) qualitative analysis of cognitive functions, in particular, the peculiarities of speech; and 3) detection of the key features that could help mark off the oddity phenomenon with regard to the method of assessment.

The design and procedure of the research sought to consolidate qualitative and quantitative methods in order to obtain the most definitive data. At the first stage of the study, the data was collected with the help of self-report measures. Then, a pathopsychological experiment was carried out, which included two steps: a non-structured clinical interview, and a set of tasks to evaluate particular qualities of cognitive abilities and speech. Finally, statistical analysis was performed, and the data was integrated and interpreted.

### Questionnaires

1. *Schizotypal Personality Questionnaire*(Raine, 1991) – SPQ-74 translated and adapted by A. Efremov and S. Enikolopov (Efremov & Enikolopov, 2001). This questionnaire was originally developed on the basis of the diagnostic criteria for the schizotypal personality disorder of DSM-III. It includes 74 items that are categorized into 9 subscales (Ideas of Reference, Social Anxiety, Odd Beliefs/Magical Thinking, Unusual Perceptual Experiences, Eccentric Behavior, No Close Friends, Odd Speech, Constricted Affect, and Suspiciousness). The items also load on three factors: 1) Cognitive Perceptual (which consists of the following subscales: Ideas of Reference/Suspiciousness, Magical Thinking, and Unusual Perceptions); 2) Interpersonal (No Close Friends/Constricted Affect and Social Anxiety); and 3) Disorganized (Eccentric Behavior and Odd Speech). They can be categorized as two poles of schizotypy: positive (subscales Ideas of Reference, Odd Beliefs/Magical Thinking, Unusual Perceptual Experiences, Odd Speech) and negative (Social Anxiety, No Close Friends, Constricted Affect, and Suspiciousness).
2. *Adult Personality Traits Questionnaire*– APTQ (Rusalov & Manolova, 2003), a self-report instrument developed on the basis of G. Shmishek’s Character Test. The APTQ includes 80 items and 10 subscales (Hyperthymia, Fixedness, Affectability, Meticulousness, Anxiety, Cyclothymia, Demonstrativity, Excitability, Dysthymia, and Emotional Reactivity).
3. *Clinical interview*(Zeigarnik, 1986). This tooka non-structured form and was aimed at establishing contact, and assessing speech patterns and magical thinking, using the criteria complementary to the similarly named subscales of SPQ-74:
  - *odd speech*– a tendency to use unusual speech constructions, where the distinctness of speech is attributable to combining words that do not usually go together, or words that belong to different registers. Also, oddity of speech included the tendency for word coinage (to create new words by combining or changing the existing ones) and the usage of words in unusual contexts. This criterion is complementary to the subscale Odd Speech of the SPQ-74, which assesses more formal characteristics of speech such as its dynamics, tempo, frequency of slippage, etc., and focuses on how understandable speech is to other people. If the aforementioned patterns of speech were detected, the patient received a score of 1 for each usage. The protocols and issued scores were then revised by an expert.
  - *magical thinking*– another criterion complementary to a similarly-named subscale in the SPQ-74, where magical thinking is evaluated in more extreme forms such as beliefs in paranormal worlds, telepathy, etc. and even includes experiencing the listed phenomena in real life. The clinical interview allowed the observation of subtler and sometimes even unconscious aspects of magical thinking, such as inconsistent religiousness and other incoherent cases (*e.g*., when a patient rationally denied believing in anything paranormal, yet later in the interview referred to his horoscope sign to explain his behavior, etc.). A binary scale was used to appoint a score of 0 or 1, according to the presence or absence of the listed characteristics. This decision was then re-assessed by another expert.
4. *Pathopsychological experiment*– a set of selected tasks aimed at assessing most aspects of thinking activity (dynamic, operational, and motivational). The tasks included a pictogram, interpretation of idioms and metaphors, and the oddball task (Rubinshtein, 2010). The psychologists’ reports of the experiment’s results were analyzed, and the distortions found within the course of the procedure were codified in order to be available for further quantitative analysis (the overall number of trials was counted, and each trial received a score of 0 or 1, depending on whether the distortion was detected or not). The distortions were registered according to the previously established criteria (Zeigarnik, 1986; Rubinshtein, 2010).

## Results

First, the clinical sample was studied as a whole, in order to investigate possible additional characteristics of the group. Surprisingly,the respondents from the clinical sample scored low on the SPQ-74 (average overall score being 19.56), and the analysis of the personal and temperamental traits of the patients revealed an average profile, without any striking peaks or declines, with the exception of a slight tendency towards the accentuation on the subscale of Dysthymia (an average score of 6.3, whereas the score of 7 would suggest the accentuation).

Then, the links between the SPQ-74 scales and temperamental and personality characteristics (assessed by APTQ) were analyzed, using the non-parametric test (Spearman’s correlation).*Table 1* shows the most relevant correlations between the subscales of the SPQ-74 and some subscales of the APTQ.

**Table 1:** Significant Correlations between SPQ-74 and APTQ subscales

	HYP	FIX	MET	CYC	DYS	EMR
**SPQ-74 (sum)**		0.40	0.43	0.48		0.56
**Lack of Close Friends**			0.41	0.42		0.49
**Restricted Affect**	-0.42		0.42		0.43	
**Suspiciousness**		0.44				
**Odd speech**				0.45		0.47
**Social Anxiety**						0.50
**Negative Schizotypy**			0.42	0.45		0.51
**Positive Schizotypy**						0.50
**F1 (Cognitive/Perceptual)**			0.45			0.47
**F2 (Interpersonal)**			0.44			
**F3 (Disorganized)**						0.46

*Note. HYP = Hyperthymia; FIX = Fixedness; MET = Meticulousness; CYC = Cyclothymia; DYS = Dysthymia; EMR = Emotional Reactivity.*

As expected, the Restricted Affect subscale showed a positive link with the Dysthymia scale and a similarly discernable negative one with the Hyperthymia scale. The Fixedness scale showed significant correlation with the Suspiciousness subscale, which allows for a more expansive interpretation of the latter. In fact, this correlation may extend and explain suspiciousness in terms of hostility, lack of empathy, unforgivingness, etc., and also imply that suspiciousness and fixedness might be mediated by stress, as suspiciousness is named among the possible coping strategies for individuals who demonstrate the trait of fixedness (Rusalov & Manolova, 2003). Another important finding was the variety of links between the characteristics of mood with a number of the SPQ-74 subscales (Hyperthymia, Cyclothymia, and Dysthymia), which suggests that emotional instability may be internally linked to the oddity phenomena.

The Emotional Reactivity scale showed the highest number of significant links with various SPQ-74 subscales. This personality feature also reflects emotional instability with mood swings, along with high reactivity and impulsivity, and tendencies towards increased impressionability and attention-getting expressiveness.

**Figure 1.**
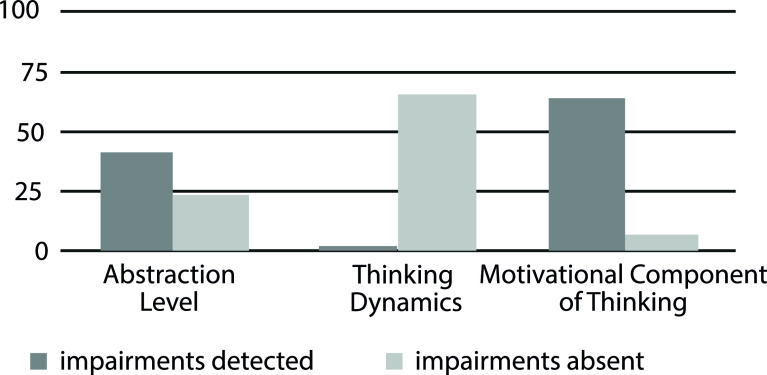
Thinking impairments in clinical sample.

The results of the analysis of the cognitive sphere of the patients with oddity phenomena are summarized in *Figure 1*. The most intact aspect of thinking in our clinical sample was the subjects’ thinking dynamics; here, the great majority of subjects (98.6%) did not show any impairment, whether it be passivity or lability of thinking processes. Even though more than half of the patients demonstrated the ability to generalize by categories (57%), less than half (37%) showed a genuinely high level of abstraction (which implied correct interpretations of idioms and metaphors); 4% showed lowered levels of abstraction with a high prevalence of situation-bounded responses. Altogether, 63.4% of the patients demonstrated a reduced level of abstraction, with impaired ability to understand figurative speech. The distortion of abstraction level and motivational deficit in thinking processes proved tobe the prevalent types of thinking impairments in these patients with odd personalities.

Apart from the basic aspects of thinking, two additional domains were assessed in the course of the clinical interview: magical thinking and odd speech. These constructs had been operationalized with the help of content analysis of the protocols and further quantification of these criteria. As hypothesized, these characteristics showed significant correlations with the SPQ-74 subscales Odd Speech (Spearman’s r=0.42, p<0.01) and Magical Thinking (r=0.58, p<0.01), which proved the validity of the complementary criteria. Yet it also demonstrated that the constructs assessed with the help of a clinical interview are not completely equivalent to their corresponding subscales content-wise, and may serve as an additional source of information.

**Figure 2.**
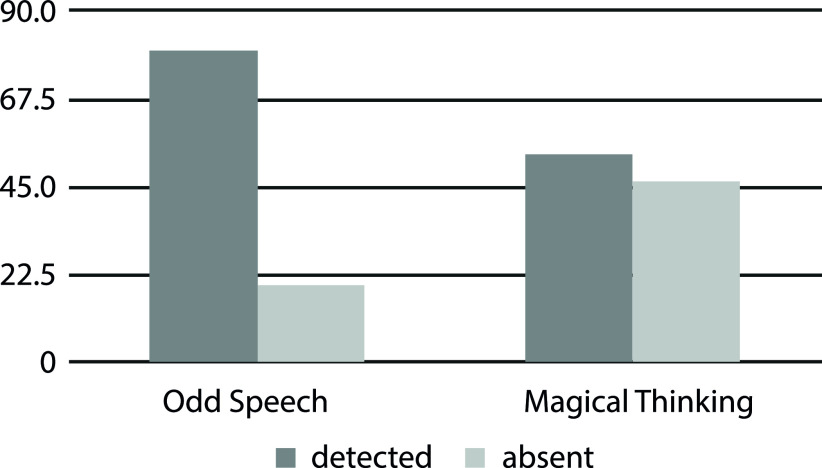
The study of complementary characteristics of thinking processes.

*Figure 2* shows that the majority of the patients with the oddity phenomenon (80.3%) tended to use unusual speech structures that fell into one or more of the following categories: a) unusual combination of words, including using the words of different register and/or in irregular context); b)word coinage; c) ornate words or phrases; and d) usage of collocations, idioms, and jargon out of the context of the situation and conversation topic. The criteria for magical thinking had also been expanded with the help of the data collected within the clinical interview – mostly by including extra characteristics that are mainly attributable to Russian culture (beliefs in superstitions, traditional medicine, etc.). Unexpectedly, only a little more than half of the patients (53.5%) demonstrated magical thinking in any of the abovementioned forms, whereas the rest of the subjects did not show any of the signs of magical thinking – a result that will be discussed in the next section.

Finally, the problem of confrontation of different “types” of oddity was addressed. The subjects’SPQ-74 scores represented self-reported oddity (how the patients – odd personalities – saw themselves), whereas the assessment of key criteria for oddity by the clinicians (with the method of clinical interview and pathopsychological experiment) constituted clinically assessed oddity. Thus, two sets of sub-groups were identified, and the SPQ profiles were then compared.

**Figure 3.**
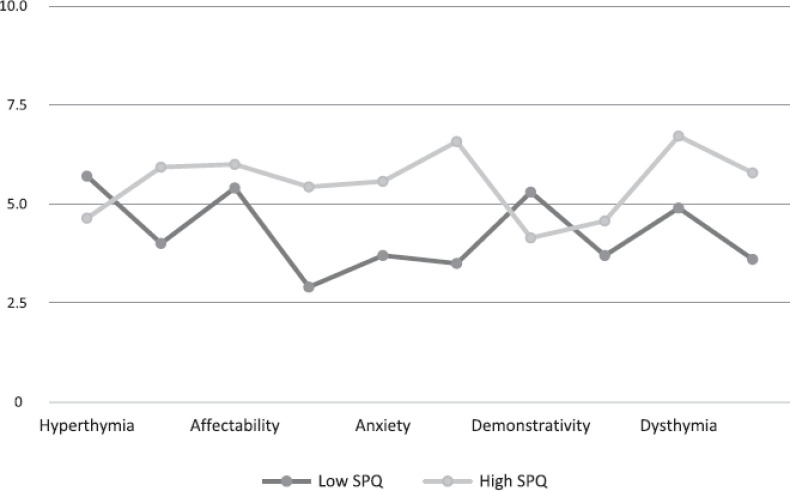
Averaged APTQ Profiles of groups with low and high SPQ-74 scores.

To determine any statistically significant differences between these “extreme” groups, we ran a Wilcoxon test, which revealed differences in the Odd Speech parameter as assessed by the clinical interviews (p=0.010), and a number of the APTQ scales: Fixedness (p=0.001); Meticulousness (p=0.016); Cyclothymia (p=0.000); and Emotional Reactivity (p=0.000). Having noted that the prevailing differences had been detected in temperamental and personality traits, we constructed a graph to illustrate the difference between the profiles of the “extremes” groups as assessed by APTQ parameters (see *Figure 3*). It demonstrated that the levels of Fixedness, Meticulousness, Cyclothymia, and Emotional Reactivity were significantly lower in patients with the lowest SPQ-74 scores. This means that patients who considered themselves less, or not at all, odd, also tended to ignore insults and/or criticism, showed certain carelessness and irresponsibility in their daily routine as well as emotional indifference and remoteness, and reported a more stable mood.

Then, based on the overall score that patients were appointed by the experts (which was assigned first by the psychologist and then re-evaluated by the council of clinicians), the sample was divided into two different groups, that is, a “less odd” group, clinically perceived as having less prominent odd traits, and, in contrast, a “highly odd” group. The criteria correlated positively and moderately with the overall SPQ-74 scores (Spearman’s r=.508). Further, the Wilcoxon test revealed that the classification of the subjects into two groups, figuratively less and more odd, based on the clinicians’ criteria, revealed significant differences on all SPQ-74 scales (p<0.020), with the exception of Restricted Affect scale of SPQ-74. These findings may indicate that the specialists’assessment differentiated between less odd and more odd personalities at least as successfully as the validated self-report measure. Further analysis showed statistical differences in such parameters of thinking as the Abstraction level (p=0.007), as well as the Emotional Reactivity Scale of APTQ (p=0.050).

## Discussion

The fact that patients with oddity phenomena scored low on the SPQ-74 may signify that schizotypal individuals, classified as such based on clinicians’ criteria, might not be aware of, or insightful about their condition. Furthermore, the general tendency of the presented profiles (SPQ-74 and APTQ) is towards averaging, which is not unsurprising in the current study. The clinical sample included individuals with rather diverse and long medical histories, which described odd, unusual behavioral patterns, as well as peculiar emotional and personality characteristics. In light of this, it was not entirely unexpected that employing self-report measures did not yield any significant results.

These findings support the idea that self-report measures are vulnerable to a number of the external influences that are widely discussed in clinical literature (Paulhus & Vazire, 2007). Consequently, in the case of the current analysis, the questionnaire does not seem sufficient for diagnosing oddity without additional means of personality assessment. Moreover, a highly averaged temperamental and personality profile may imply that no specific profile can be developed which coherently defines the oddity phenomenon, at least not based solely on the common personality and temperamental traits assessed by APTQ. This finding had been expected since none of specific personality types, apart from schizotypal itself (which had been initially derived from the observed peculiarities), can be held accountable for an individual’s appearance to be characterized as “odd” or “strange.”

The analysis of the interrelations between a number of the APTQ parameters and the SPQ-74 subscales revealed a range of traits that may be particularly important for some schizotypal characteristics in the clinical sample. We found that a general instability of mood and a tendency for inadequate and/or paradoxical emotional reactions, as well as the predominance of a particular extreme of mood (that is, melancholy with signs of anxiety, passivity, etc., or excitability with high activity level, ingenuity of judgment, and so forth), were the most prominent features. Another distinguishing characteristic was rigidness in behavior, judgment, and affect, which manifested through commitment to inner principles, lack of empathy, and high levels of aspiration. It can be hypothesized that inflexibility, tendencies to develop predominant ideas or interests, low emotionality, and focus on personally significant goals might create an image of oddity for such individuals and their consequent lack of engagement.

Emotional Reactivity is also a characteristic that was found to have high importance to the schizotypal style. It is described in the official manual of ATPQ as the “inability to conceal one’s feelings” (Rusalov & Manolova, 2003, p. 86), and, since such traits as emotional lability and general expressiveness cannot be controlled sufficiently, they can contribute to seemingly inadequate, or socially unacceptable, behavior.

The analysis of the cognitive sphere revealed that the thinking process of patients with the oddity phenomenon was characterized by the impairment of its motivational aspect and a tendency to generalize based on latent characteristics. According to traditional and modern clinicians, these patterns are more characteristic of schizophrenia-spectrum disorders, including schizotypy. Thus, these findings may indirectly support the modern view of the oddity phenomenon as pertaining to the spectrum of negative disorders, as hypothesized in the recent work by Russian clinicians (Smulevich et al., 2017). On the other hand, these results can also indicate the tendency of clinicians to employ some symptoms of motivational impairments in thinking (*e.g.,* tangential thinking, or cognitive slippage) as inconspicuous signs of oddity itself. Further studies are needed in order to determine whether the detected cognitive impairments are essentially related to the diagnoses, or is simply part of the “odd” image.

Further analysis of their speech showed that the great majority of the patients demonstrated at least one of the designated signs of unusualness in speech patterns, which may imply that speech peculiarities are a significant feature of the oddity phenomena. Undoubtedly, the use of the specific structures in speech as described in the previous section will facilitate the impression of one’s speech as strange and inappropriate, and also serve as an implicit sign of social insensitivity – a tendency to ignore conventional standards of communication (Kritskaya & Meleshko, 2015; Morrison, Brown, & Cohen, 2013; Zeigarnik, 1989). In particular, bizarrerie, or affectation of style, which term describes a number of thinking and speech patterns that are cumbersome and loaded with irrelevant details (Cherednikova, 2015; Zeigarnik, 1989; Zhmurov, 2012), was found to be prominent in the studied sample. Therefore, such complexity makes speech too difficult to comprehend and generally evokes a sensation of theatricality and/or constraint. Bizarrerie is also considered to be a specific sign of schizophrenic spectrum disorders (Cherednikova, 2015; Panteleeva, Cucul'kovskaya, & Belyaev, 1986), as well as characteristic of a schizotypal personality (APA, 2013).

When it comes to the assessment of magical thinking in patients with oddity, cultural norms must be taken into account, since most Russian-speaking groups, including mentally healthy individuals, have demonstrated more or less high levels of magical thinking (Bairamova & Enikolopov, 2016). Researchers attribute this to Russian culture, which normalizes magical thinking in a variety of forms (*e.g*., beliefs in superstitions, traditional medicine, etc.). In this connection, we introduced a complementary criterion in the course of the clinical interviews, which allowed exploration of not just beliefs in the paranormal, but also incongruence between declared and real beliefs (where real beliefs tend to be subconscious or concealed but in reality determine individual’s behavior). However, this additional criterion did not result in qualifying magical thinking as a prevailing feature in the clinical sample, which led us to question the diagnostic significance of this particular characteristic – at least in this specific Russian-speaking sample.

Lastly, since our research focused only on one kind of clinical sample – that is, individuals with confirmed medical histories and long-established odd personalities – we addressed the juxtaposition between the self-reports and clinically assessed oddity. For these purposes, we divided the sample in two different sets of so-called “extreme” groups: 1) the two groups with the lowest and the highest SPQ-74 scores; and 2) two alternative groups qualified by the experts as “the most” and “the least” odd according to the relevant clinical criteria, evaluated with the help of qualitative methods and then revised by a council of experts consisting of both medical doctors and psychologists.

The two criteria showed a moderately strong positive correlation (r=.508), and the division based on the clinical criterion was also confirmed by the corresponding significant differences in SPQ-74 scores. In other words, when patients reported themselves as odd, it only partially coincided with the clinicians’ assessment, but when clinicians rated some individuals more odd than others, it turned out to be mostly congruent with the self-report data.

These findings suggest that both ways of measuring the oddity phenomena are more likely to assess the same phenomenon than not, yet the differences between these methods should definitely be accounted for. Altogether, the differentiation between the “extreme” self-report groups appeared mainly to be related to some parameters of emotionality (Cyclothymia and Emotional Reactivity) as well as personality traits, whereas the chief focus of the specialists seemed to be just emotionality and cognitive characteristics. Obviously, the parameters of cognitive functioning are not likely to be assessed in any kind of self-report measure; yet they may have importance for identifying and studying oddity.

## Conclusion

The notion that the individuals who are perceived as odd and eccentric may form a special group that can be defined by a consistent complex of manifestations, has been the focus of many researchers since the beginning of the 20th century. Later, as this notion developed, it was interpreted in the context of the schizotypy construct, where oddity was given a central role. To avoid conceptual confusion and to focus attention solely on the oddity phenomena, we selected a clinical sample with a long-established history of expressed oddity, and included criteria concentrated on the relevant characteristics of all mental spheres instead of a formal diagnosis of schizotypal personality disorder, since the latter could have allowed unwanted heterogeneity.

The profiles of schizotypal (as assessed by the SPQ-74) and temperamental and personality traits (as assessed by APTQ) were constructed based on data from the entire sample, and their tendency towards the average was revealed, which is supportive of the view of schizotypy as a singular, independent construct which requires more qualitative analysis than self-report measures can provide.

The qualitative analysis of the cognitive sphere, including speech, revealed a range of peculiarities and impairments that pertain to the cluster of symptoms specific to schizophrenia-related disorders. However, whether these findings add to the evidence of schizotypy being placed on the spectrum of negative disorders, or simply uncover the method of clinical assessment of oddity, is yet to be investigated.

The question of differentiation between self-reported and clinically assessed oddity has been raised. We found that both methods of evaluation are of importance; however, clinical assessment naturally encompasses more spheres of psychological functioning and generally provides more insight into the psychology of odd personalities, since the latter tend to ignore, block, or remain otherwise unaware of their peculiarities.

Finally, in addition to a validated measure of schizotypal traits, new means of measurement and assessment have been employed and analyzed, which could pave the way for further investigation of the oddity phenomenon.

## Limitations

The discussion of whether particular personality types can be specifically tied to the oddity phenomenon indeed requires further research. Due to the limited availability of the respondents of our clinical sample, there were restrictions on the number of methods that could be applied, and a single self-report measure of the personality sphere combining both common personality traits as well as major temperamental characteristics was required. Future research will need to take into account a generally accepted typology of personality types and focus on the interconnections between more personality features and the peculiarities of cognitive sphere.

One of the chief problems we identified was with using self-report measures, such as SPQ-74, to study schizotypy in patients with the oddity phenomenon. The absence of significant differences between the scores of the clinical sample and the normative data may be attributed to widely acknowledged weaknesses of self-report measures in general (Paulhus & Vazire, 2007), as well as the specific nature of the oddity phenomenon. Whether or not this problem is exclusive to this particular group of patients, or relevant for other groups as well, is open to discussion and requires further research.

Finally, even though the current study could not fully support the hypothesis that magical thinking, as defined by the SPQ-74 and complemented by additional criteria, is particularly likely to be responsible for an individual giving the impression of “oddity,” further research for evidence in favor of this hypothesis needs to be done, since the literature suggests that magical thinking is still a relevant part of the characteristic features of schizophrenia-spectrum disorders, and schizotypy in particular.
